# On the Comparative Merits of Cow-Pock and Small-Pox Inoculation

**Published:** 1806-04

**Authors:** C. B. Trye

**Affiliations:** Surgeon to the Infirmary at Gloucester


On the Comparative Merits of Cow-Pock and Small-Pox Inoculation; *by* C. B. Trye, *Surgeon to the Infirmary at Gloucester.*

I HAVE lately read some extraordinary publications, written by Messrs. Rowley and others, hostile to vaccination; extraordinary indeed, for the present day, in point of composition, reasoning, and narrative.

I do not think that they will influence any mind, which is in the habit of scrutinizing evidence, argument, or authorities; but as multitudes (and amongst them many worthy and estimable individuals) are not in that habit, at least in respect to medical subjects; I request you, for their sakes, to print the following remarks, which, brief as they are, I verily believe, contain an antidote equal to the poison which these papers may infuse.

First of all, they bring forward a number of cases, some of them probably true, in which vaccination, apparently well conducted, has failed to be a security against the small-pox. I have given some instances already*, and I could give a great number of others, in which inoculation with small-pox matter has been equally insecure.—Be it remarked then, first, that it does not appear that any *universally certain, absolute,* and *indefeasible* preservative against the small-pox has ever yet been obtained. At the same time it *does* appear, that so *near* an approach to that *universality* has been obtained, that any individual may be as fully assured, *for ever,* against the small-pox, as he can at any time be assured of his life for a single day.

Secondly; there are two ways of obtaining this assurance against small-pox; by inoculation, and by vaccination.

X 3 That

---

* See Medical Journal, Vol. xii. p. 396.

That the latter is at least equally efficacious with (probably more than*) the former, the experience of this country bears a *general* testimony; and the experience of every other portion of the civilized earth bears an *universal* testimony.

Thirdly; as to the *comparative safety* of the two expedients; in this county, within the last seven years, to my knowledge, great numbers have been vaccinated, and I know that *not one of those has died*, or been in the *least danger* of dying *from vaccination.*

Within the preceding seven years, great numbers, in this same county, have been inoculated with small-pox. Of these, *I know* that some have died, and many more been in imminent danger of dying *from inoculation.* My understanding, then, cannot hesitate in pronouncing vaccination, or artificial cow-pox infection, infinitely the *safer* expedient.

Fourthly; as these gentlemen dwell upon the dreadful effects of vaccination, let us consider the consequences of the two diseases—cow-pox and small-pox.

The consequences are to be divided into two sorts; the *certain* and *immediate*, and the *doubtful* and *remote.* Let it be observed, that the disease produced by small-pox inoculation, in no essential whatsoever, differs from that which is taken, as it is called, *naturally*; and that the cow-pox, by vaccination, is precisely the same as the cow-pox caught *accidentally*, as in the dairies. The *certain* and *immediate consequences* of small-pox, when the disease has been very severe, (I speak as an eye-witness) have been incurable blindness, from pustules being formed in the eyes themselves, mortifications on the back and on the nates, extensive abscesses and ulcers, general and lasting debility, and what is no slight evil, the *change of beauty for deformity*. Now the disease, in its utmost malignity, is occasionally the sequel of inoculation, as well as of the natural infection. But among great numbers who have been vaccinated, within the reach of my observation, not the most trifling inconvenience has once occurred *immediately*, and *certainly* consequent on vaccination.

As *remote and doubtful ill consequences* of small-pox, whether inoculated or natural, I have witnessed scrophula, enlarging and ulcerating the glands, and producing white swellings, obstinate ophthalmias, with entire loss of the eye-lashes, varieties of herpes, &c. Among those who have

* See Mr. Child's experiment in the postscript.

have been vaccinated, I have seen none of these ill consequences (nor, indeed, any others), excepting herpes, which were imputed, or imputable to vaccination. But after all, it is extremely questionable (and therefore I have made use of the term doubtful), if the evils which I have noticed as the remote consequences of small-pox, were properly the offspring of small-pox. It is rather to be conceived that the disposition producing them pre-existed in the habit, and that small-pox acted no further, than merely exciting (as any other powerful agent would have done) that disposition; and in *like manner* we must speak of any diseases which we may see spring up apparently from the effect of vaccination, if, contrary to the experience of this neighbourhood, such should spring up. But I do not believe, that any of those distressing complaints, which I have mentioned, as the remote or doubtful cosequences of small-pox, will be found to be in like manner the consequences of vaccination, because of the inference which necessarily flows from the following certain facts.

The first I shall insist upon is, that a more healthy description of human beings does not exist, nor one more free from chronic cutaneous impurities, than that which suffers most from cow-pox by reason of their being employed in dairies.

The second, that the Gloucester Infirmary, one of the largest provincial hospitals, is situated in a county in which accidental cow-pox has been prevalent from time immemorial: many hundreds, among the labouring people, have had that cow-pox since the establishment of this institution, and *that, more severely than is generally the case in artificial vaccination*; and yet *not a single patient, in half a century, has applied to the infirmary for the relief of any disease, local or constitutional, which he or she imputed, or pretended to trace to cow-pox.* And be it repeated and remembered, that the *artificial* in no respect differs from the *accidental* cow-pox, except in being generally less virulent. The consequences, therefore, whether immediate or remote, cannot differ, the cause, and the circumstances in which that cause acts, being the same.

As for what these writers say about one horrible consequence, to wit, the transformation of the human countenance into that of an ox; I will not insult the understandings of your readers, by endeavouring to expose the absurdity of this matter now, or to remove apprehensions of the like in future.

One word more, on the *peculiar conveniency* of vaccination.

tion. Admitting that societies of persons, who have never had small-pox, may contain individuals, of whom fears may be entertained, as to the consequences of vaccination; and whom, notwithstanding, it may be inconvenient to divide from their associates, still the vaccination of the others may go on, without risque of these exceptionable subjects catching the disease. Moreover, among those, to whom labour and intercourse with the world are necessary, both may be continued without injury, either to themselves or others, during vaccination; which cannot be said of, *nor ought to be permitted in*, small-pox.

I conclude with earnestly pressing these reflections (especially what I have said upon the infirmary) on those minds, which have been, by any means, rendered fearful of, or prejudiced against, the cow-pox; humbly hoping, that they may be instrumental in disposing them to co-operate with the greatest and wisest of our countrymen, and with every government in Europe, in rooting out the small-pox; a work, which will, in all human probability, be shortly accomplished, if the practice of vaccination be not obstructed.

*Gloucester*, *Feb.* 16, 1806.

*P. S.* The respectability of Mr. Birch's professional situation, induces me to treat his queries * with particular attention. To the second query, "Does the puncture of small-pox inoculation ever produce such inflammation of the arm as to kill the patient?" The answer must be in the affirmative, as will appear from the following extract of a letter from Mr. Williams, late a very respectable and experienced surgeon in Dursley, now retired from business, and therefore a most disinterested evidence.

"Mr. ——— underwent inoculation for the small-pox. The arm went on very well till the sickening fever came on, when it became inflamed, tumefied, and very painful. A sphacelus succeeded, which terminated speedily in death."

A similar inference may be drawn from Dr. Rowley's Answer to the Committee of the House of Commons. See Report of the Committee.

To the paragraph which follows the queries in Mr. Birch's letter, all I have to say is, I sincerely lament that a per-

* See Mr. Birch's letter to Mr. Rogers.

a person placed in the honorable station which Mr. Birch has filled during so many years, could prevail upon himself to write it; and this whether we consider either its liberality or its good sense.

The gentleman above noticed, Mr. Williams, communicated also in writing the following case.

"A man had three children successfully inoculated with variolous matter. On their recovery he came to me, complaining of head-ach, vertigo, nausea, sickness, violent pain in the small of his back, and wandering pains all over him, attended with considerable fever. He observed, that *if he had not had the small-pox,* he should think he was going to sicken for it. I replied, that his face (which was pitted to a very great degree) was a perfect security against such an apprehension. On which he said, that he had the small-pox very badly twenty years ago, when he was about sixteen.—The next day a number of little red spots appeared on the skin, which increased daily till they came to a certain size, and then suppurated to the very basis; in which state the patient was seen by the late Dr. Hardwick of Sodbury, who observed, that at this rate, if people were to have the small-pox twice, there might as well be an end of inoculation.

Mr. Child, surgeon, of Northleach, made the following clear and decisive experiment. A woman had a large burden of small-pox; she had an infant who lay with her, about two years old. Mr. Child inoculated one arm of the infant with small-pox taken from its mother, the other arm with cow-pox immediateley afterwards.

The small-pox arm inflamed in the usual manner, and on the fifth day, the inflammation was advanced as far, as it usually is at that period, and had the customary appearance; from that time it began to decline, and died away altogether on the seventh day, nor had the child any subsequent variolous symptom. The vaccinated arm went completely through its usual stages.

A most terrifying account having been giving of mortifications seizing upon the extremities, in consequence of vaccination; *without altogether denying the veracity of the narration,* perhaps, what follows may quiet the alarms, which such narration may have excited.

Children, of peculiar constitutions, are remarkably subject to chilblains, and will be seized with them, even in warm weather. Among the poorer classes, it is not uncommon for this species of inflammation to run into gangrene, or into phagedenic ulcer, which I have repeatedly seen seen destroy one toe after another; and the ravages of which, were only to be stopped by the greatest care, and the most generous diet. In bulky children, whose complexions were uncommonly pale, I have twice seen a small discoloration in the cheek degenerate into gangrene, which, neither medicine, nor any other means, were able *to repress*, and which, in a few days, destroyed the patient. The following case will farther illustrate the above observation.

The 2d of December last, I vaccinated a reputable tradesman's child, about a year old; it was very fat, but of that uncommon pallid complexion that I hesitated, and was only persuaded to inoculate it from the circumstance of the small-pox being at the next door. The progress of the infection was much as usual, except that the areola was less florid. About the fourteenth day, the father told me, that the child had been in great pain the whole of the preceding night; that two days before, it had scratched a finger with a pin, and that the finger was now *mortifying*. I found the whole hand swollen, and, apparently, having a chilblain. The fore finger, upon the last joint, had a *black blister*, as large as half a horse bean; the child had been in excessive pain, with sickness, and a very quick small pulse. I wrapped the finger in lint, wetted with laudanum, and the hand in sal ammoniac, vinegar, and spirit of wine, and prescribed syrup of white poppies freely, and extract of bark. The mischief was speedily corrected, but an ulcer was formed upon the finger, which, like other ulcers arising from chilblains, was slowly, and difficultly healed.

